# Impact of an evolving classification system on diffuse glioma repositories: experience from the Sydney brain tumour bank

**DOI:** 10.1007/s11060-026-05470-1

**Published:** 2026-02-19

**Authors:** Laveniya Satgunaseelan, Elissa Xian, Daniel Madani, Kasuni K. Gamage, Susannah M. Hallal, Vineet Gorolay, Hao-Wen Sim, Sofia Mason, Michael E. Buckland, Brindha Shivalingam, Kimberley L. Alexander

**Affiliations:** 1 Department of Neuropathology, Royal Prince Alfred Hospital, Camperdown, NSW Australia; 2 Faculty of Medicine and Health, University of Sydney, Sydney, NSW Australia; 3 Department of Neurosurgery, Chris O’Brien Lifehouse, Camperdown, NSW Australia; 4 Department of Radiology, Royal Prince Alfred Hospital, Camperdown, NSW Australia; 5 Department of Radiology, Royal North Shore Hospital, St Leonards, NSW Australia; 6 Department of Medical Oncology, Chris O’Brien Lifehouse, Sydney, NSW Australia; 7 Faculty of Medicine and Health, University of New South Wales, Sydney, NSW Australia; 8 Garvan Institute of Medical Research, Darlinghurst, NSW Australia

**Keywords:** CNS tumors, Cancer registry, Glioma, Genomics, Methylation profiling

## Abstract

**Purpose:**

Brain tumour classification is a rapidly evolving field, with diagnostic evaluation integrating the latest in molecular testing techniques. As data in brain tumour registries and repositories are collected in real time, neuro-oncology researchers face clear challenges when analysing tumour cohorts diagnosed according to differing standards over time. This study aims to evaluate the impact of an evolving tumour classification system on both our institutional registry and widely used multi-institutional repositories in glioma translational research.

**Methods:**

Clinicopathological data, including molecular profiles, were obtained from the Sydney Brain Tumour Bank registry (1993–2025). We sourced available clinicopathological and molecular classification data from the Rembrandt and Gravendeel datasets, the Chinese Glioma Genome Atlas (CGGA) and The Cancer Genome Atlas (TCGA). All cases were reclassified according to the WHO Classification of Tumours of the Central Nervous System (5th edition).

**Results:**

Between 37% and 100% of cases diagnosed prior to the 2016 WHO Classification (revised fourth edition) require additional molecular testing for accurate diagnosis and grading. In contrast, the majority of cases in datasets established after the 2016 Classification met the WHO 2021 Classification criteria (61% to 97%). Two cohorts that consistently failed to meet 2021 requirements over time were high-grade gliomas in patients under 55 years and histological grade 2/3 IDH-mutant gliomas.

**Conclusion:**

An evolving tumour classification system necessitates regular review and reclassification of brain tumour datasets to ensure that brain cancer research is accurate and equitable. The reclassified datasets are provided for use by neuro-oncology researchers worldwide.

**Supplementary Information:**

The online version contains supplementary material available at 10.1007/s11060-026-05470-1.

## Introduction

Biobank registries are real-world datasets that rely on precise diagnostic classification for clinically meaningful translational research [1, 2]. Analysing tumour cohorts diagnosed according to varying standards can lead to significant pitfalls in data interpretation and in correlating with patient outcomes [3]. Of all tumour types, brain tumour diagnosis, particularly glioma, has seen the most marked change in diagnostic classification over the past two decades [4]. Given the use of several key registries in translational brain cancer research [5–8], the effect of this evolution on the utility of these datasets warrants evaluation.

Prior to the 2007 World Health Organisation (WHO) Classification of Tumours of the Central Nervous System (CNS), immunohistochemical (IHC) techniques and fluorescent in situ hybridisation (FISH) were emerging as diagnostic adjuncts [9]. By the time the 2016 WHO Classification was published, IDH mutation status and 1p/19q codeletion assessment were established as clinically important diagnostic indicators [10]. FISH became a commonly used technique for evaluating 1p/19q codeletion, alongside single-gene sequencing for non-canonical *IDH1/2* mutations [11, 12]. The arrival of the 2021 Classification heralded an expanded range of molecular biomarkers for diagnostic stratification, necessitating the adoption of next-generation sequencing (NGS) and other technologies (e.g. chromosomal microarray) to test all genomic alterations [13]. The most notable changes within this Classification included the introduction of prognostic molecular markers for the grading of adult-type diffuse gliomas (e.g. *CDKN2A/B* homozygous deletion for IDH-mutant gliomas; molecular features of glioblastoma, IDH-wildtype, including *TERT* promoter hotspot variants, *EGFR* amplification and combined chromosome 7 gain and 10 loss); a deeper genomic characterisation of paediatric-type glioma (e.g. histone H3-mutant gliomas); and the ushering in of methylation profiling as a key tool in the neuropathologist’s diagnostic armamentarium [13]. Brain tumour diagnosis in the current era requires integrating multiple data elements, including clinical information (age, anatomical location), histopathology, genomic findings, and methylation profiling to arrive at an integrated diagnosis [4].

The Sydney Brain Tumour Bank has been in operation since the early 2000s, collecting not only brain tumour tissue but also patient-reported survey data on quality of life and post-treatment symptoms, electronic health record (eHR) data including treatment records, survival outcomes, and postmortem data from brain autopsies. As the WHO Classifications evolved, our group observed that older diagnoses collected contemporaneously from the eHR did not necessarily align with the most current version of the Classification. We also noted that several widely used datasets (Rembrandt, Gravendeel, Chinese Glioma Genome Atlas [CGGA], The Cancer Genome Atlas [TCGA]) have been similarly affected by an evolving classification scheme. In this paper, we aim to quantify the effect of the 2021 WHO Classification requirements on data held in multi-site glioma biobanks and data registries.

## Methods

### Data collection and cohort selection

We accessed data from 707 glioma cases (primary and recurrences) collected from 514 patients by the Sydney Brain Tumour Bank between 1993 and 26 August 2025 (Supplementary Table 1), including patient demographics, treatment records, and complete pathology work-ups, including molecular testing performed at the time (SLHD HREC X19-0010). We grouped SBTB cases according to periods defined by successive WHO Classifications:

1. pre-2007 (FISH introduced),
2. WHO 2007 (2007 to 2016; IHC, FISH and *IDH1/2* pyrosequencing introduced),
3. WHO 2016 (2016 to 2021; NGS panel introduced, with *CDKN2A/B* NGS testing added towards the end of this period), and
4. WHO 2021 (post-2021; methylation profiling introduced – DKFZ Heidelberg Classifiers v11b4, v12.5 and v12.8 were used depending on the version available at the time of diagnosis).

For the repositories investigated alongside SBTB data, exclusion criteria included cases lacking a primary diagnosis or those designated as ‘non-tumour’ without accompanying molecular testing. The Rembrandt, Gravendeel and CGGA phenotypic data were accessed from the GlioVis portal (http://gliovis.bioinfo.cnio.es/, accessed 23 September 2024) [14]. TCGA data was accessed from cBioPortal (cbioportal.org, accessed 23 September 2024). The Rembrandt dataset [8] included 444 eligible cases profiled between 2004 and 2006 (pre-2007; Supplementary Table 2). The Gravendeel dataset [7], published in 2009 after the WHO 2007 Classification, included 278 eligible cases (Supplementary Table 3). The CGGA [6] (1010 eligible cases from RNASeq_1018 dataset) and TCGA [5, 15–17] (1047 eligible cases from TCGA-GBM and TCGA-LGG) datasets were accrued and published between the 2016 and 2021 WHO Classifications (Supplementary Tables 4 and 5). Each case in these repositories is from a unique patient.

### Classification status

We developed a coding system to assess the adequacy of diagnosis and grading in accordance with WHO 2021 requirements (Table 1).

Over time, further delineation of the association between patient age and the diagnosis of high-grade glioma has emerged both within the 2021 Classification and from the neuropathology literature.

1. Patients <55 years: gliomas in this group should be investigated for non-canonical *IDH1/2* mutations[13];
2. Patients ≤50 years: testing for histone H3 mutations, particularly at the p.G35 hotspot, has diagnostic utility[18];
3. Patients <40 years: molecular testing for diffuse paediatric-type high-grade glioma, H3-wildtype, and IDH-wildtype should be considered [19, 20].

**Table 1:** Coding system used to denote adequacy of diagnosis and grading against the WHO 2021 requirements

Abbreviation	Meaning
U	Diagnosis remains unchanged
N	Diagnosis does not require further molecular testing but nomenclature change required
Dx	Diagnosis requires further molecular testing for diagnosis
Gx	Diagnosis requires further molecular testing for grading
Dx Gx	Diagnosis requires further molecular testing for diagnosis (Dx) and/or grading (Gx)
Y	Tumour that can be classified on the basis of testing of a matched tumour
N (R)	Primary tumours that achieved the WHO 2021 Classification based on the testing of a matched tumour recurrence but require nomenclature change
U (R)	Primary tumours that achieved the WHO 2021 Classification based on the testing of a matched tumour recurrence but require no nomenclature change
Unk	Diagnosis remains unclear after full work-up, including methylation profiling
(?) IDHm UG astro	Previously histological grade 2 IDH-mutant (IDHm) astrocytoma without *CDKN2A/B* copy number testing (UG = ‘ungradable astrocytoma’)
(?) IDHm HG astro	Previously histological grade 3 IDH-mutant astrocytoma without *CDKN2A/B* copy number testing (HG = ‘high-grade astrocytoma’)
(?) IDHm UGG	Previously histological grade 2 IDH-mutant gliomas without 1p/19q codeletion status and *CDKN2A/B* copy number testing (UGG = “ungradable glioma”)
(?) IDHm HGG	Previously histological grade 3 or 4 IDH-mutant gliomas without ATRX or 1p/19q codeletion status (HGG = “high-grade glioma”)
(?) UGG NOS	Previously histological grade 2 gliomas lacking relevant molecular testing for definitive classification and grading
(?) HGG NOS	Previously histological grade 3 or 4 gliomas lacking relevant molecular testing for definitive classification and grading, with particular respect to patient age and anatomical location

Abbreviations: IDHm = IDH-mutant; astro = astrocytoma; UGG = “ungradable glioma”; HGG = “high-grade glioma”; NOS = not otherwise specified

## Results

### Pre-molecular era of morphological diagnosis (pre-2007)

**Fig. 1:**
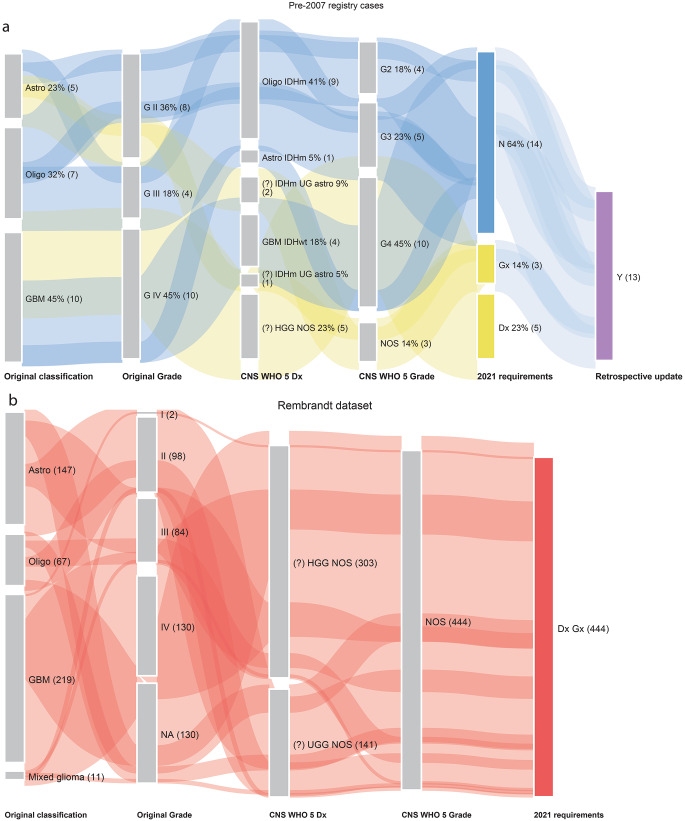
(**a**) Sankey plot of SBTB pre-2007 registry cases, re-classified to meet WHO 2021 Classification requirements. (**b**) Sankey plot of Rembrandt dataset, re-classified to meet WHO 2021 Classification requirements. Abbreviations: astro = astrocytoma; oligo = oligodendroglioma; GBM = glioblastoma multiforme; GII / III / IV = grade 2 / 3 / 4; oligo IDHm = oligodendroglioma, IDH-mutant and 1p/19q-codeleted; astro IDHm = astrocytoma, IDH-mutant

The pre-2007 SBTB (*n* = 22) and Rembrandt (*n* = 444) datasets contain no unchanged diagnoses (‘U’) (Fig. 1); all cases required either additional molecular testing or a nomenclature revision. Cases requiring nomenclature change (‘N’) without further molecular testing were primarily oligodendroglioma, IDH-mutant and 1p/19q-codeleted (previously known as ‘oligodendroglioma’), astrocytoma, IDH-mutant, CNS WHO grade 4 (all of which were histologically grade 4; previously termed ‘secondary glioblastoma’ or ‘glioblastoma, IDH-mutant’) and glioblastoma, IDH-wildtype (GBM IDHwt; previously named ‘glioblastoma multiforme’).

The Rembrandt dataset includes only histological diagnoses; the absence of pertinent molecular alterations introduced in later WHO Classifications resulted in ‘Dx Gx’ designations for all cases (Supplementary Table 2). In contrast, the pre-2007 SBTB cohort benefits from the introduction of FISH testing (*n* = 10), and retrospective updates following the molecular testing of matched tumour recurrences (*n* = 13). Notably, five initially diagnosed GBMs in this cohort were reclassified as ‘GBM IDHwt’ (*n* = 4) and ‘astrocytoma, IDH-mutant (n = 1), CNS WHO grade 4’, changes that affected nomenclature only within the biobank registry. The ‘Dx’ category consisted entirely of ‘HGG NOS’ cases, all of which have age-related molecular testing requirements. The ‘Gx’ category comprised ‘IDHm UG astro’ cases that lacked *CDKN2A/B* testing.

### Towards the molecular era with the introduction of basic molecular techniques (2007–2016)

**Fig. 2:**
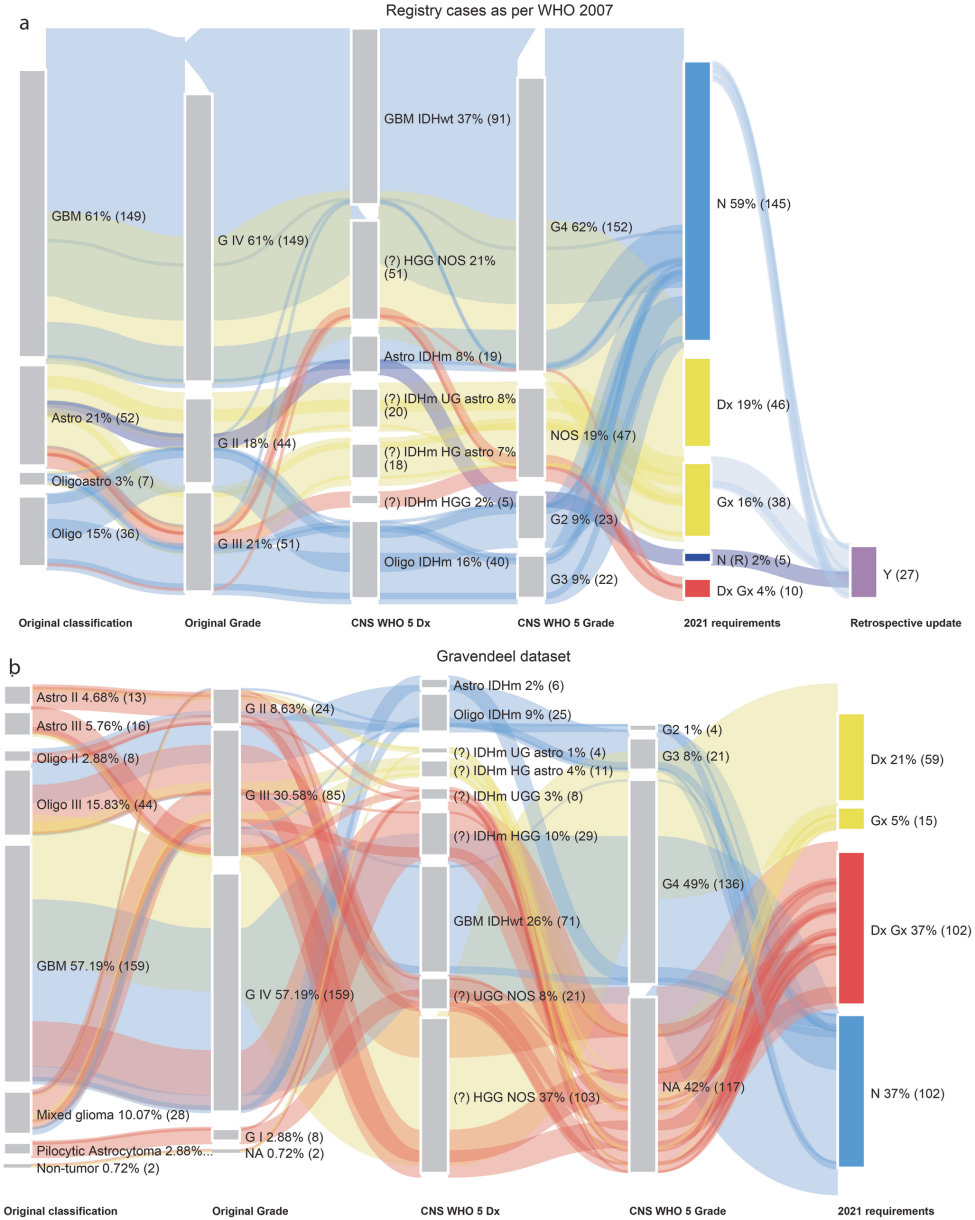
(**a**) Sankey plot of SBTB WHO 2007 registry cases (2007–2016 period), re-classified to meet WHO 2021 Classification requirements. (**b**) Sankey plot of Gravendeel dataset, re-classified to meet WHO 2021 Classification requirements. Abbreviations: oligoastro = oligoastrocytoma

The WHO 2007 SBTB cohort (*n* = 244) contained a higher proportion of cases that were either unchanged or required only nomenclature revision (‘N’= 59%, ‘N(R)’=2%) compared with the Gravendeel dataset (‘N’=37%) (Fig. 2). As before, the ‘N’ group comprised oligodendroglioma, IDH-mutant and 1p/19q-codeleted, astrocytoma, IDH-mutant, CNS WHO grade 4 and GBM IDHwt. The two cases designated ‘N(R)’ were IDH-mutant astrocytomas in which *CDKN2A/B* copy number testing had been performed on matched recurrent tumour specimens.

The Gravendeel dataset (*n* = 278) included *IDH1* status in 80% of cases (*n* = 223) but lacked *IDH2* variants analysis, and reported 1p/19q codeletion status for 48% (*n* = 134) of the cohort (Supplementary Table 3). Overall, 64% of Gravendeel cases (‘Dx’ + ‘Gx’ + ‘Dx Gx’, *n* = 176) required additional molecular testing to meet WHO 2021 criteria.

Within the WHO 2007 SBTB cohort, 38% of cases (‘Dx’ + ‘Gx’ + ‘Dx Gx’, *n* = 99) required further molecular testing. Although molecular testing (*IDH1/2* sequencing and/or FISH for 1p/19q codeletion) was not performed in 67% of these cases, IDH1 R132H IHC was assessed in the majority (89%; *n* = 216). This is particularly relevant for GBM IDHwt, where, according to WHO 2021 criteria, a diagnosis can be established based on a combination of patient age, tumour location, and IDH1 R132H immunonegativity. Additionally, 11% of cases (*n* = 27) could be retrospectively reclassified based on molecular testing of matched recurrent tumours.

### The advent of the molecular era (2016–2021)

**Fig. 3:**
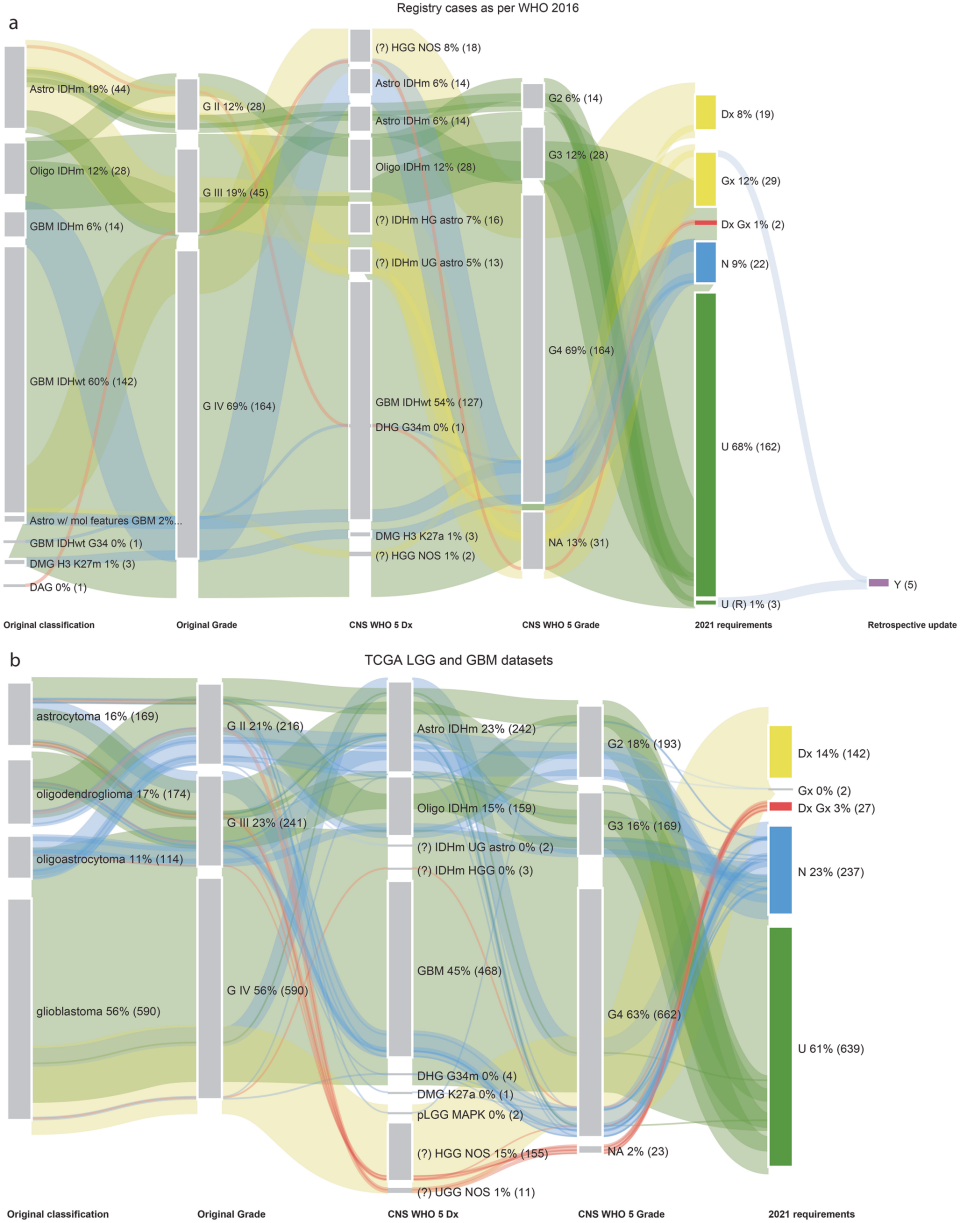
(**a**) Sankey plot of SBTB WHO 2016 registry cases (2016–2021 period), re-classified to meet WHO 2021 Classification requirements. (**b**) Sankey plot of TCGA LGG and GBM datasets, re-classified to meet WHO 2021 Classification requirements. Abbreviations: GBM IDHm = glioblastoma, IDH-mutant; GBM IDHwt = glioblastoma IDH-wildtype; astro w/ mol features of GBM = diffuse astrocytic glioma with molecular features of GBM; GBM IDHwt G34 = glioblastoma, H3 G34 mutant; DMG H3 K27m = diffuse midline glioma, H3 K27-mutant; DAG = diffuse astrocytic glioma; DHG G34m = diffuse hemispheric glioma, H3 G34-mutant; DMG, H3 K27a = diffuse midline glioma, H3 K27-altered

As expected, most cases in both the WHO 2016 SBTB (*n* = 237) and TCGA cohorts (*n* = 1047) had either unchanged diagnoses or required nomenclature revision (‘N’ + ‘U’, WHO 2016 SBTB = 77%, *n* = 184; ‘N’ + ‘U’, TCGA = 84%, *n* = 876) (Fig. 3). The difference between cohorts is primarily attributable to the ‘Gx’ category in the WHO 2016 SBTB dataset, which, as in earlier periods, was comprised of IDH-mutant astrocytomas (histological grades 2 and 3), as our institution had not yet introduced *CDKN2A/B* testing. In contrast, this biomarker was captured via whole-genome sequencing (WGS) in the TCGA dataset (Supplementary Table 4). Interestingly, new entities recognised in the 2021 WHO Classification began to emerge during this period, including diffuse midline glioma, H3 K27-altered; diffuse hemispheric glioma, H3 G34-mutant; and diffuse paediatric low-grade glioma, MAPK pathway-altered. High-grade astrocytoma with piloid features (HGAP), a rare entity defined exclusively by its DNA methylation profile, was also introduced [13]. In the absence of methylation profiling, HGAP is difficult to distinguish from other high-grade gliomas. As the WHO diagnostic criteria were applied strictly to other high-grade gliomas, it is possible that the ‘HGG NOS’ category includes some HGAP cases.

The CGGA cohort (*n* = 1010) showed similar proportions of ‘U’ and ‘N’ cases to the Gravendeel datasets (‘U’= 32%, ’N’=9%), and 22% categorised as ‘Dx Gx’ (n = 218) (Supplementary Fig. 1a). Most of these were IDH-wildtype cases, with further classification constrained by the absence of testing for defining molecular features of GBM IDHwt (Supplementary Table 5). The ‘Dx Gx’ cases in the TCGA cohort were similarly composed of mostly IDHwt tumours; however, WGS did not identify molecular features diagnostic of GBM IDHwt. These cases would likely benefit from additional testing with methylation profiling. In the WHO 2016 SBTB cohort, limited tissue availability for molecular testing restricted definitive classification of ‘Dx Gx’ cases.

### The molecular era in full flight (post-2021)

**Fig. 4:**
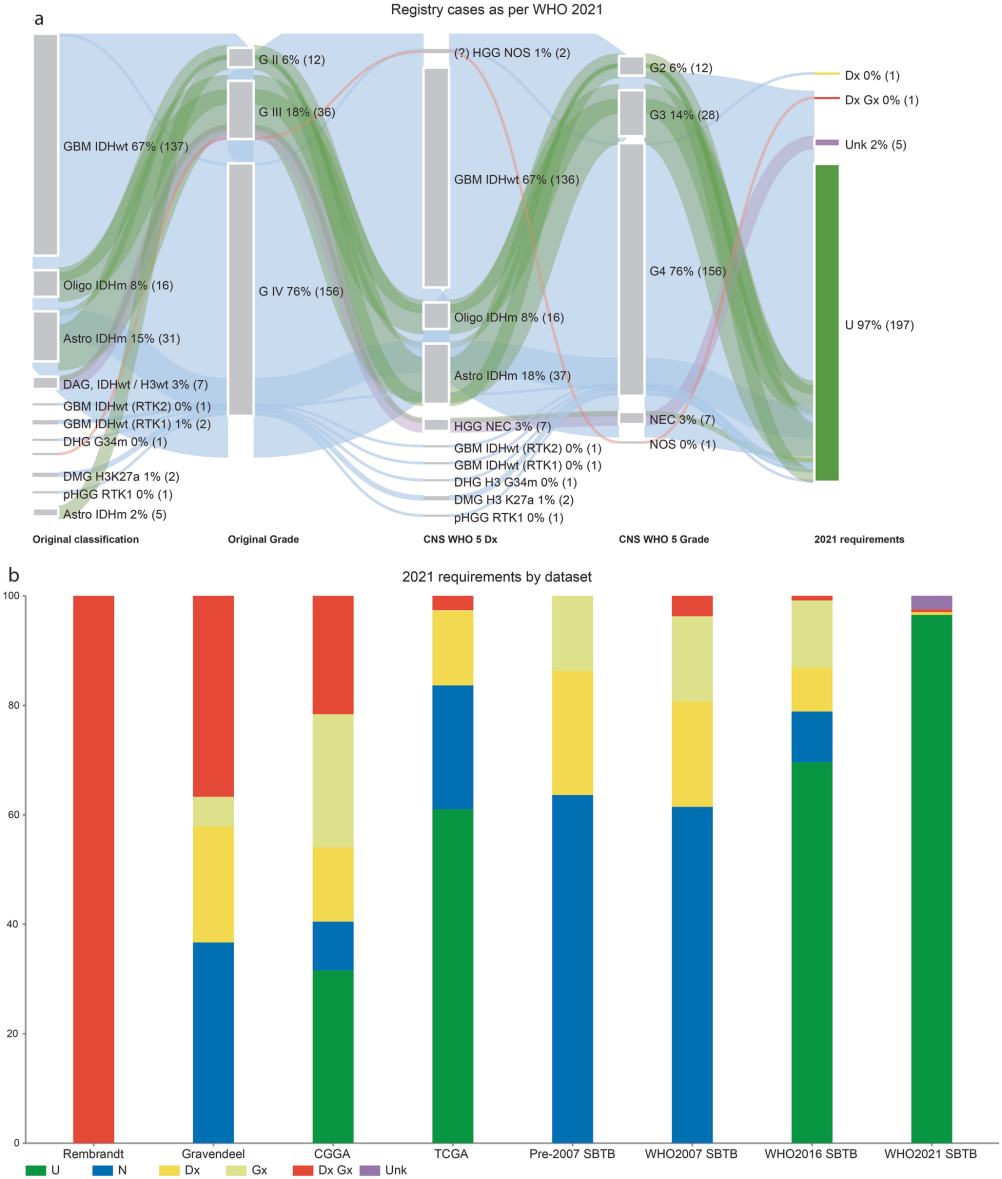
(**a**) Sankey plot of SBTB WHO 2021 registry cases (2021 – August 2025 period), as per WHO 2021 Classification requirements. (**b**) Requirements for WHO 2021 Classification requirements by dataset. Abbreviations: pHGG RTK1 = diffuse paediatric-type high-grade glioma, H3-wildtype and IDH-wildtype, RTK1 subtype; HGG NEC= high-grade glioma, not elsewhere classified; GBM IDHwt (RTK2)= glioblastoma, IDH-wildtype, RTK2 subtype; GBM IDHwt (RTK1)= glioblastoma, IDH-wildtype, RTK1 subtype

Following publication of the WHO 2021 Classification, the majority of SBTB cases fulfilled diagnostic criteria, with several notable exceptions (Fig. 4a). In addition to newly defined genomic entities, classification increasingly reflected methylation profiling results, identifying subtypes such as GBM IDHwt RTK1 and RTK2, and pHGG RTK1 subtype. Five diffuse gliomas, each histologically grade 3 and IDH- and H3-wildtype, remained unclassifiable despite NGS and methylation profiling. All cases had at least 80% tumour cellularity, and their methylation profiles, though inconclusive, excluded non-neoplastic or control tissue classes. These cases, therefore, remain with a descriptive diagnosis, ‘not elsewhere classified’.

Residual ‘Dx Gx’ and ‘Dx’ designations in the WHO 2021 SBTB cohort were primarily due to insufficient tissue for the full suite of molecular testing.

### The effect of an evolving CNS WHO classification over time

Figure 4b illustrates the cumulative impact of the WHO 2021 Classification on both international repositories and the Sydney Brain Tumour Bank.

Regarding prognostic biomarkers, the number of cases requiring molecular testing for definitive grading (‘Gx’) decreased over time. The ‘Gx’ category comprises IDH-mutant astrocytomas or gliomas, histologically grade 2 or 3, for which *CDKN2A/B* copy number testing is unavailable. Testing for molecular features of GBM IDHwt in histologically grade 2 or 3 IDH-wildtype diffuse gliomas did not contribute to the ‘Gx’ category. In the TCGA dataset, WGS enabled the assessment of all relevant molecular features and the assimilation of these cases into the GBM IDHwt category. Similarly, in the WHO 2016 SBTB cohort, four cases previously described as ‘astrocytoma with molecular features of GBM’ were reported after the introduction of NGS panel testing in our laboratory [21]. The Gravendeel dataset included testing for a single GBM IDHwt-associated molecular feature (*EGFR* amplification), identifying four cases of ‘molecular GBM’. For the remaining IDH-wildtype diffuse gliomas, the absence of GBM IDHwt molecular marker testing necessitated additional molecular analysis for definitive diagnosis and grading (‘Dx Gx’), recorded as ‘UGG NOS’ or ‘HGG NOS’.

Regarding diagnostic biomarkers, the number of cases requiring molecular testing for definitive diagnosis (‘Dx’) also decreased over time. The ‘Dx’ category consisted entirely of ‘HGG NOS’ cases initially diagnosed as ‘GBM’. All patients in this group were under 55 years of age (Supplementary Fig. 1b), an age range that now triggers additional molecular testing for definitive classification.

As molecular testing became increasingly comprehensive, the proportions of ‘N’ and ‘U’ cases rose accordingly. Nomenclature-only revisions first appeared from 2007 onward and included oligodendroglioma, IDH-mutant and 1p/19q-codeleted, astrocytoma, IDH-mutant, CNS WHO grade 4 (where grade was assigned on histology), and GBM IDHwt. These three entities have maintained relatively stable combinations of histological features and molecular features that can be resolved using basic molecular techniques such as IHC and FISH.

## Discussion

Brain tumour classification has adopted molecular testing techniques more rapidly and with greater agility than other organ systems [22]. Most CNS tumours now require molecular testing for diagnostic classification, enabling greater prognostic accuracy and refined treatment selection [23]. This diagnostic precision has also driven therapeutic discovery, exemplified by the recent approval of vorasidenib for low-grade IDH-mutant gliomas [24]. IDH mutations were among the earliest genomic variants identified in gliomas, and their molecular characterisation has led to the development of one of the first targeted therapies for patients with brain tumours [24–26]. The advent of molecular profiling has fundamentally reshaped the WHO Classification of CNS tumours, a transformation of enduring significance.

Because gliomas are rare in the general population [27], translational glioma research has historically relied on large, multi-institutional repositories to aggregate data to achieve sufficient statistical power and meaningful biological insights [5–7]. Many of these datasets remain in use by researchers today [28–30], including some that predate the incorporation of IDH status into glioma classification. Additionally, when reclassification affects grading, survival estimates may be affected, with implications for clinical trial design. It is therefore critical that the limitations of these datasets be rigorously evaluated, particularly within the context of an evolving diagnostic framework, to ensure that biologically disparate entities are not conflated. In this study, we examined the impact of successive WHO Classification updates on these datasets and on our own institutional registry.

We first observe that histological classification alone, as utilised in the Rembrandt dataset, is no longer sufficient to stratify patients, even into low-grade glioma and GBM [28]. There is now clear evidence that glial neoplasms with a histologically low-grade appearance may harbour molecular alterations that warrant reclassification as high-grade tumours [13, 31, 32]. Similarly, ‘GBM’ can no longer be regarded as a monolithic entity. At a minimum, IDH status should be determined, as reflected in the 2021 WHO Classification, which recognises ‘astrocytoma, IDH-mutant, CNS WHO grade 4’ as a distinct entity from GBM [13].

To this end, IHC is an important screening tool for repository and registry datasets. For example, under the current WHO 2021 criteria, a diagnosis of GBM IDHwt can be made if the patient is 55 years or older, has a tumour in a non-midline location, has no history of a prior lower-grade glioma, in which IDH1 R132H immunohistochemistry is negative [13]. In the SBTB cohorts, retrospective confirmation of GBM IDHwt diagnoses from 2007 was possible because IDH1 R132H IHC was routine.

IHC is a cost-effective alternative to more advanced molecular testing platforms and can be readily applied to archival formalin-fixed, paraffin-embedded (FFPE) tissue, which is often the only material available in legacy registry cohorts. For histone mutant gliomas, H3 K27M and H3 K27me3 IHC are integral to the workup of diffuse midline glioma, H3 K27-altered, with loss of H3 K27me3 being an essential diagnostic criterion [13]. In contrast, IHC for H3 G34 variants is less reliable than for H3 K27M and IDH1 R132H, and sequencing remains recommended for confirmation [33].

Similarly, surrogate IHC markers for key prognostic genomic alterations are still emerging. There is growing evidence that loss of MTAP and p16 IHC staining reliably reflects *CDKN2A/B* copy number status [34–37]. When only limited or FFPE tissue is available, IHC markers like these help bridge classification gaps in biobank registries. In large repository datasets, however, IHC markers are more difficult to implement; when WGS or whole-exome sequencing data are available, retrospective analysis could facilitate re-evaluation of these biomarkers.

Our study revealed that tumours with relatively stable combinations of histological features (e.g. histological grade 4) and molecular features detectable by basic molecular techniques (IHC or FISH) stood the test of time. These included oligodendroglioma, IDH-mutant and 1p/19q-codeleted, astrocytoma, IDH-mutant, CNS WHO grade 4 (where grade was assigned on histology) and GBM IDHwt. However, not all high-grade gliomas could be reliably classified using basic techniques, and further diagnostic refinement was required, particularly regarding patient age. While testing for non-canonical *IDH1*/2 variants has become increasingly common in patients under 55 years, testing for histone H3 hotspot variants in patients under 50 years was not routinely performed prior to the 2021 Classification. More recently, cIMPACT-NOW Update 11 has recommended testing for paediatric diffuse high-grade glioma, IDH- / H3-wildtype in patients under 40 years, a diagnosis that often necessitates methylation profiling [19]. Although methylation profiling data are now being compiled in registry datasets [38], including TCGA and CGGA, such data are absent from some of the repository datasets evaluated here. The implications of this are evident in our study, where cases of HGAP may be present among diagnoses labelled ‘HGG NOS’. The lack of contemporary molecular profiling may disproportionally disadvantage adolescent and young adult glioma research, limiting diagnostic accuracy and impeding meaningful cross-study comparability.

This raises a critical question: how can brain tumour registries be designed to remain robust and relevant amid the continual evolution of classification frameworks? Within our institutional cohort, we explored whether recurrent tumours, tested using more advanced techniques than were available at the time of primary diagnosis, could inform retrospective classification. This approach proved most valuable in the pre-2007 SBTB cohort, where IDH1 R132H IHC and FISH performed on recurrent specimens enabled refined classification of oligodendroglioma, IDH-mutant and 1p/19q-codeleted and GBM IDHwt. As methylation profiling and other advanced techniques emerge, testing of recurrent tumours may increasingly help refine or confirm primary tumour classifications. However, molecular data derived from recurrent specimens, particularly prognostic biomarkers, must be interpreted within their full clinical, radiological and histopathological context. To ensure that research based on these resources remains accurate and comparable, registry and repository datasets may need to undergo periodic reclassification as diagnostic frameworks continue to evolve.

By reclassifying several well-known and widely used international repositories, we demonstrate that specific datasets (Rembrandt [8] and Gravendeel [7]) should be interpreted with caution, as they lack the granularity required to study homogenous tumour cohorts. In contrast, the more recent datasets, i.e., TCGA [5] and CGGA [6], can be utilised effectively, provided the revised nomenclature (see Supplementary Information) introduced by the WHO Classification is used. We note two recently published studies that sought to re-classify TCGA dataset [39, 40]. Problematically, these attempts did not take into account the anatomical location (accessible via cBioPortal [15–17]), histone H3 status, and *CDKN2A/B* status, emphasising the critical importance of expert neuropathology input to ensure accurate tumour classification and brain tumour registry maintenance. Beyond molecular testing, registries must be mindful of changes in disease ontology. For example, pilocytic astrocytoma was previously designated as International Classification of Diseases for Oncology (ICD-O) code M9421/3, indicating a malignant behaviour code [41]. However, this has since been revised to ICD-O-M9421/1 (borderline/non-malignant code) to align with its established clinical behaviour [41, 42]. As molecular profiling improves in prognostic accuracy, further changes to disease ontology systems are expected and will be reflected in registry data where possible.

Within our own registry, we recognise the advantage of alignment with evolving WHO molecular testing requirements, largely enabled through close integration with our institution’s neuropathology department. This alignment supports accurate tumour classification at diagnosis and facilitates reclassification as diagnostic criteria evolve. We recommend that new registries collect highly granular histopathological and molecular testing data to maintain flexibility and long-term validity. Nevertheless, even with comprehensive updates, a subset of tumours remains unclassifiable on retrospective review. We also acknowledge that many low- and middle-income countries in our region may lack access to advanced molecular technologies needed to update their registries, an issue currently being addressed by the Asian-Oceanian Society of Neuropathology [43, 44].

All reclassified datasets are provided in the Supplementary Information for the benefit of the global brain cancer research community, to support accurate tumour classification and to enable meaningful translational studies [45]. The datasets can also serve as well-matched comparators or external control arms for single-arm or hybrid trials, improving eligibility matching and supporting treatment decisions and guideline updates [46]. 

## Supplementary Information

Below is the link to the electronic supplementary material.

Supplementary Material 1

Supplementary Material 2

Supplementary Material 3

Supplementary Material 4

Supplementary Material 5

Supplementary Material 6

## Data Availability

All datasets generated and/or analysed during the current study are available in the Supplementary Information. Further details of the Sydney Brain Tumour Bank registry are available from the corresponding author on reasonable request.
