# Supplementary figures and images for: Impact of an evolving classification system on diffuse glioma repositories: experience from the Sydney brain tumour bank
Source: J Neurooncol. 2026 Feb 19;177(1):6. doi: 10.1007/s11060-026-05470-1 (PMC12920384; doi:10.1007/s11060-026-05470-1)

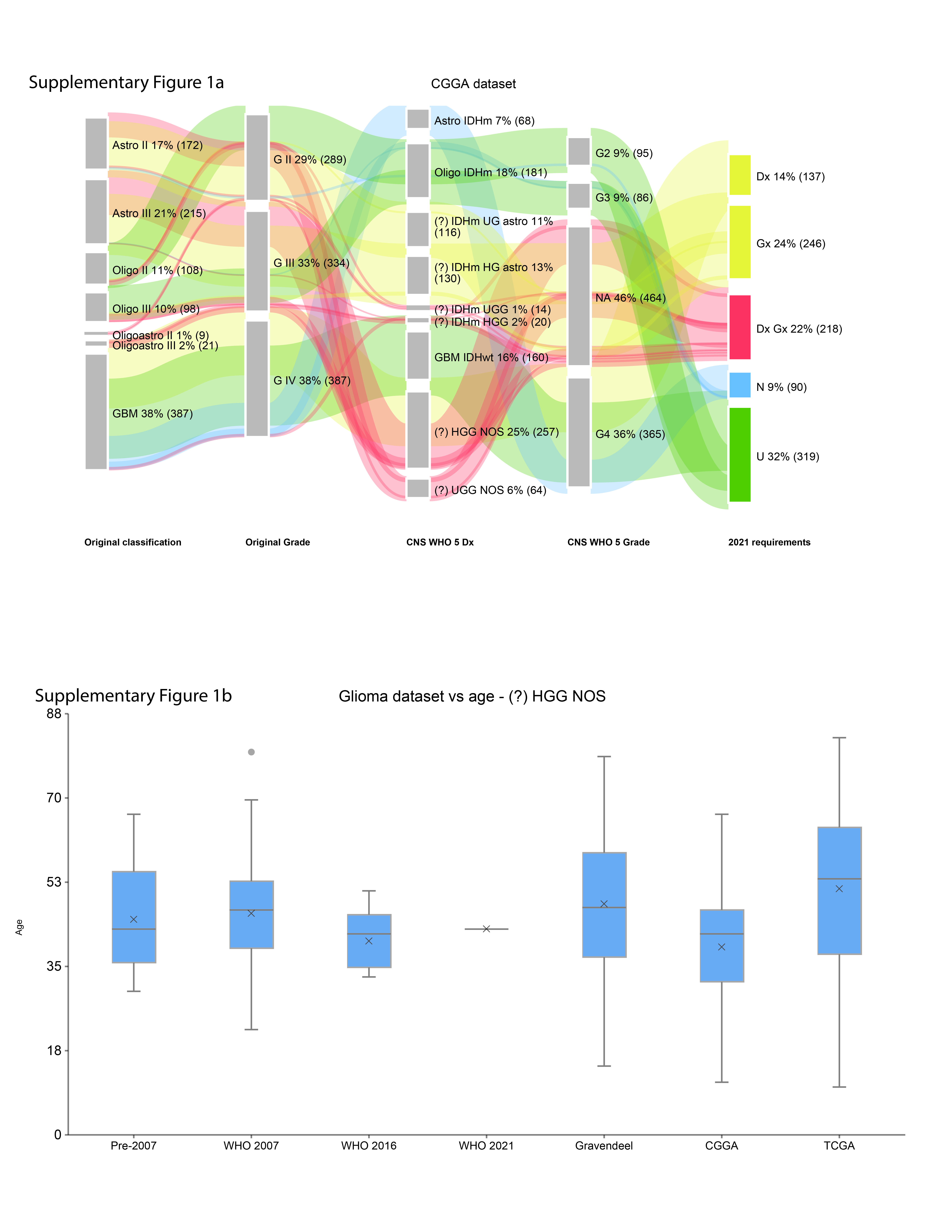

Supplement: Supplementary file 5 — Supplementary Material 5 [file 11060_2026_5470_MOESM5_ESM.jpg]
